# Formulation Optimization of a Palm-Based Nanoemulsion System Containing Levodopa

**DOI:** 10.3390/ijms131013049

**Published:** 2012-10-11

**Authors:** Syafinaz Zainol, Mahiran Basri, Hamidon Bin Basri, Ahmad Fuad Shamsuddin, Siti Salwa Abdul-Gani, Roghayeh Abedi Karjiban, Emilia Abdul-Malek

**Affiliations:** 1 Department of Chemistry, Faculty of Science, Universiti Putra Malaysia, 43400 UPM Serdang, Selangor, Malaysia; E-Mails: salwa@science.upm.edu.my (S.S.A.-G.); rosa.abedi@gmail.com (R.A.K.); emilia@science.upm.edu.my (E.A.-M.); 2 Laboratory of Molecular Biomedicine, Institute of Bioscience, Universiti Putra Malaysia, 43400 UPM Serdang, Selangor, Malaysia; 3 Department of Medicine, Faculty of Medicine and Health Science, Universiti Putra Malaysia, 43400 UPM Serdang, Selangor, Malaysia; E-Mail: hamidon@medic.upm.edu.my; 4 Centre for Drug Delivery Research, Faculty of Pharmacy, Universiti Kebangsaan Malaysia, Jalan Raja Muda Abdul Aziz, 50300 Kuala Lumpur, Malaysia; E-Mail: afsna@pharmacy.ukm.my; 5 Quality Use of Medicines Research Group, Faculty of Pharmacy, Universiti Kebangsaan Malaysia, Jalan Raja Muda Abdul Aziz, 50300 Kuala Lumpur, Malaysia

**Keywords:** palm oil, nanoemulsions, levodopa, Response Surface Methodology, central composite design

## Abstract

Response surface methodology (RSM) was utilized to investigate the influence of the main emulsion composition; mixture of palm and medium-chain triglyceride (MCT) oil (6%–12% *w*/*w*), lecithin (1%–3% *w*/*w*), and Cremophor EL (0.5%–1.5% *w*/*w*) as well as the preparation method; addition rate (2–20 mL/min), on the physicochemical properties of palm-based nanoemulsions. The response variables were the three main emulsion properties; particle size, zeta potential and polydispersity index. Optimization of the four independent variables was carried out to obtain an optimum level palm-based nanoemulsion with desirable characteristics. The response surface analysis showed that the variation in the three responses could be depicted as a quadratic function of the main composition of the emulsion and the preparation method. The experimental data could be fitted sufficiently well into a second-order polynomial model. The optimized formulation was stable for six months at 4 °C.

## 1. Introduction

Parkinson’s disease (PD) is one of the central nervous system (CNS) diseases which is expected to rise with increasing lifespan and population demographics in the future. Various types of drugs are used to treat and relieve the symptoms of the disease including levodopa, ropinirole, apomorphine and selegiline. However, levodopa is the “gold standard” for anti-parkinsonian therapy and consequently, nearly every PD patient eventually receives this drug [1].

At present, 95% of all new potential therapeutics has poor pharmacokinetics and biopharmaceutical properties [2]. Therefore, it is necessary to produce efficient drug delivery systems in which the drug molecule reaches only the target site of action, without influencing other organs and tissues. Nanotechnology plays an important role in therapies of the future as nanomedicines could lower doses required for efficacy as well as increasing the therapeutic indices and safety profiles of new therapeutics [3]. The drug delivery systems include liposomes, micelles, nanoemulsion, nanoparticulate systems and dendrimers.

Nanoemulsion systems are potential carriers for efficient delivery of drugs across the blood-brain barrier. In general, they are biocompatible, biodegradable, physically stable (particularly nanoemulsion and microemulsions) and relatively easy to produce on a large scale using proven technology [4]. Their non toxic and non irritant nature makes them ideal therapeutic agents as they do not damage human and animal cells [5]. Their long term physical stability confers an additional advantage. Nanoemulsion in parenteral delivery has been adopted for supplying nutritional requirements, controlled drug release, vaccine delivery and for drug targeting to specific sites [6]. Intravenous administration of nanoemulsions is shown to be very advantageous, particularly due to their droplet size of less than 1 μm [7]. In addition, the emulsions can minimize the pain associated with intravenously administrated drugs by exposing the tissue to lower concentrations of the compounds [4].

Palm oil is yielded from the fruit of the *Elaeis guineensis* tree. As a vegetable oil, palm oil is one of the promising resources of industrial fats and oils due to its various beneficial properties such as high thermal, high productivity and oxidative stability. Palm oil has been widely used in the food industry. It has not been explored in pharmaceutical applications. Palm oil for pharmaceutical application should be explored and expanded due to its favorable properties such as a long chain triglyceride, nontoxicity and low cost. In order to meet some application requirements such as pharmaceutical and cosmetic formulations, there is a need to convert the oil into an emulsion. In the preparation of palm oil emulsion, organic solvents are not needed. However, until now, there does not seem to have been any report on the use of palm oil in an emulsion formulation for drug delivery.

Response Surface Methodology (RSM) is a tool which consists of mathematical and statistical techniques which are derived from the fit of empirical models to the obtained data from experiments. In order to explain the studied system, linear or square polynomial functions are utilized. Hence, the experimental conditions can be investigated for the optimization study. RSM has been used broadly to develop and optimize new formulations as it can evaluate all potential factors simultaneously. From the experimental design, the influence of the formulation variables on responses can be determined and the effects of factor interaction can be investigated. The advantages of using RSM are reported to be the reduction in the number of experimental runs needed to evaluate multiple variables and the ability of the statistical tool to identify interactions [8]. Therefore, it is less laborious and time consuming compared to studying one-variable at a time [9]. Additionally, this experimental methodology generates a mathematical model which is presented in graphical form [10].

In this work, levodopa was selected as the targeted drug to be loaded in the nanoemulsion as it is widely used to treat and relieve the symptoms of PD. To date, levodopa is only available for the patient in tablet form. Therefore, the aims of this work by using RSM were to formulate an optimal novel palm-based nanoemulsion containing levodopa and to evaluate simultaneously the main effects and interaction effects between the factors including composition of oil, lecithin and Cremophor EL as well as the addition rate on the responses; particle size, zeta potential and polydispersity index.

## 2. Results and Discussion

### 2.1. Screening of Variables

A preliminary study was carried out to evaluate the levels of independent variables. Based on the resultant data, the lower, middle and upper levels of the four independent variables were determined. Levodopa nanoemulsions showed particle size below 200 nm, narrow size distribution and zeta potential of more or less ±25 mV by restraining the range of oil, lecithin, Cremophor EL composition and addition rate at levels of 6%–12%, 1%–3%, 0.5%–1.5% and 2–20 mL/min, respectively.

### 2.2. Fitting the Response Surface Models

The variation in the particle size, polydispersity index and zeta potential were predicted by employing response surface methodology as the responses were the function of the emulsion composition and preparation variables of levodopa loaded nanoemulsions. Table 1 shows the experimental data obtained for the three response variables based on central composite design (CCD) matrix. The experimental data was statistically analyzed. The statistic analysis was used to determine the best fitted model for the four independent variables. The estimated regression coefficients, *R*^2^, adjusted *R*^2^, regression (*p*-value), lack of fit (*p*-value) and probability values related to the effect of the four independent variables are shown in Table 2. A positive value in the regression equation represents an effect that favors optimization due to a synergistic effect, while a negative value indicates an inverse relationship or antagonistic effect between the factor and the response [11]. It should be mentioned that non-significant (*p* < 0.005) linear terms were included in the final reduced model if quadratic or interaction terms containing these variables were found to be significant (*p* < 0.05) [12].

In this work, the response surface analysis demonstrated that the second-order polynomial used for particle size has a higher coefficient of determination (*R*^2^ = 0.9757) compared to the polydispersity index (*R*^2^ = 0.9632) and the zeta potential (*R*^2^ = 0.9093). The obtained coefficient of determination showed that more than 90% of the response variation of the particle size, zeta potential and polydispersity index could be described by RSM models as the function of the main nanoemulsion and preparation variables. It was observed that the lack of fit had no indication of significant (*p* < 0.05) for the final reduced model, therefore proving the satisfactory fitness of the response surface model to the significant (*p* < 0.05) factors effect (Table 2).

From Table 2, it was observed that only two independent variables (C and D) exhibited a positive effect on the response of particle size (*R*_1_). For zeta potential (*R*_2_), three independent variables (A, B, C) presented a positive effect while for polydispersity index (*R*_3_) all independent variables presented negative effects except for one variable (D). Coefficients with more than one factor, or higher order terms in the regression equation, represent the interaction between terms or the quadratic relationship, respectively which suggest a non-linear relationship between factors and responses [13]. In this condition, factors can produce a different degree of response than is predicted by the regression equation if they are varied at different levels or more than one factor is changed simultaneously [11]. All the responses *R*_1_, *R*_2_ and *R*_3_ were affected by the interaction of independent variables, presenting a quadratic relationship. The interaction effects between A and B and between A and D were favorable only for response *R*_2_. A favorable effect was also noticed for all responses, *R*_1_, *R*_2_ and *R*_3_ for the interaction between B and C. The interaction between B and D was favorable only for *R*_1_. However, it was observed that the interaction between A and C and between C and D had an inverse effect for all responses. Quadratic effects of all four independent variables were noticed for all the responses. The highest and positive quadratic effect for all variables was noticed for *R*_1_ while the highest and negative quadratic effect was noticed for *R*_2_.

The coefficient significance of the quadratic polynomial models was evaluated by using Analysis of Variance (ANOVA). For any of the terms in the models, a large *F*-value and a small *p*-value indicated a more significant effect on the respective response variables [14]. Table 3 shows the effect of independent variables on the variation of the physicochemical properties of levodopa-loaded nanoemulsions. The independent variables that most affect the particle size of the nanoemulsion for the linear term were lecithin composition, followed by the linear term of addition rate; the other two linear terms (oil and Cremophor EL composition) did not indicate any significant effect (*p* > 0.05). The quadratic term of lecithin composition also had a significant effect (*p* < 0.05) on the particle size of nanoemulsions. Conversely, the effect of the other three quadratic terms was insignificant (*p* > 0.05). Furthermore, the interaction between oil and lecithin composition and between oil composition and addition rate showed a significant effect (*p* < 0.05) on the particle size of nanoemulsions.

The variable which exhibited the largest effect on the zeta potential of the nanoemulsion for the linear term was Cremophor EL composition. The other three variables (oil composition, lecithin composition and addition rate) showed insignificant effects. The quadratic terms of Cremophor EL composition, addition rate and oil composition exhibited significant effects on the zeta potential as well. The interaction between oil composition and addition rate showed a significant effect on the zeta potential compared to the other interactions.

For the polydispersity index, the linear and quadratic term of the addition rate had the most significant effect (*p* < 0.001), followed by the linear term of Cremophor EL composition and interaction between Cremophor EL composition and addition rate. Thus, it was indicated that in evaluating the response variation of the polydispersity index, it was important to consider the Cremophor EL composition and the addition rate.

### 2.3. Response Surface Analysis

In general, there is a high demand in the pharmaceutical industry for the production of nanoemulsions with a smaller droplet size (<1 μm). Due to the nano-sized and kinetically stable characteristics, nanoemulsions are very efficient in encapsulating and/or solubilising the drugs and can successfully deliver them to the targeted part of the body. Direct contact of the drug with the body fluids and tissues can be avoided and the drug is released slowly over a prolonged period of time, which may lead to minimization of side effects [15–17].

**Figure 1:**
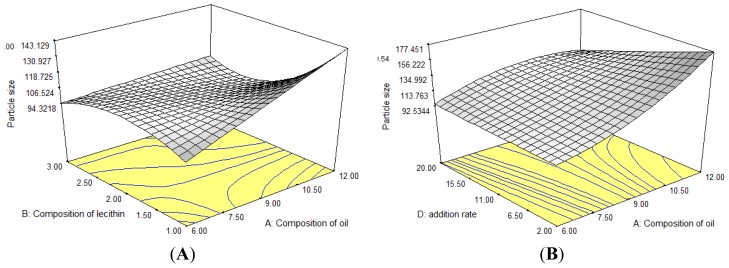
Response surface plots showing the interaction effects of (**A**) lecithin composition and oil composition as well as (**B**) oil composition and addition rate on response. *R*_1_, particle size.

**Figure 2:**
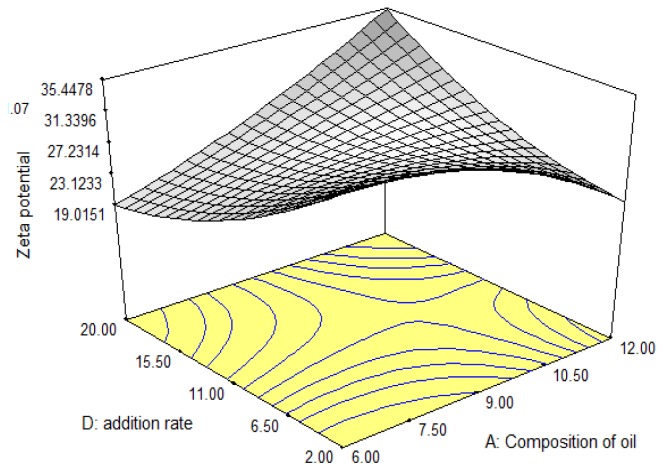
Response surface plot showing the interaction effects of oil composition and addition rate on response R_2_, zeta potential.

**Figure 3:**
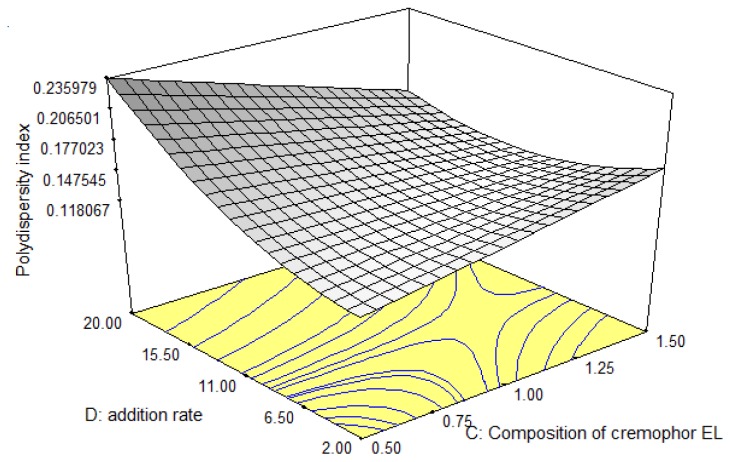
Response surface plot showing the interaction effects of Cremophor EL composition and addition rate on response *R*_3_, polydispersity index.

For the optimization of levodopa-loaded nanoemulsions, response surface analyses were plotted in three dimensional model graphs. The response surface plots for particle size, zeta potential and polydispersity index which are used to interpret the interaction effect of the variables are presented in Figures 1–3 respectively. The third and forth factors were kept at constant level. Figure 1A,B demonstrated that the particle size increases with increasing oil composition. The oil phase composition influences the physicochemical properties and the stability of parenteral lipid emulsions [18]. A few explanations need to be considered to describe the observed results. First, with the rise in the oil content, the droplet disruption process becomes more difficult which is due to an increase in the flow resistance and hence the droplet break-up rate becomes severely restricted [19,20]. Second, part of the effect can be attributed to the increased rates of collision frequency, particularly at lower concentration, between the emulsion droplets followed by an ultimate increase of coalescence frequency which subsequently lead to a higher probability of coalescence of the droplets [21].

As shown in Figure 1A, the increasing in lecithin content led to an increase in particle size. However, additional increase in the lecithin composition resulted in a decrease of the particle size. The increase in particle size may result from an impoverishment of the surfactant at the interface with increasing surface of the dispersed oil phase [20]. In addition, the increase in particle size may also be due to an insufficient amount of lecithin to emulsify the oil and the aqueous phase. Decrease of particle size by further increase in lecithin is due to the fact that the emulsifier plays a vital role in the formation of emulsion as it lowers the interfacial tension, thereby the Laplace pressure, *p* is reduced and the stress required for droplet deformation is reduced [21]. Figure 1B demonstrates increasing particle size when the oil composition is increased (9%–12% *w*/*w*) and the addition rate is reduced. Generally, particle size was reduced when the addition rate was decreased. However, the phenomenon did not occur in this case. The possible reason for this observation might be due to the relatively higher oil content. The addition rate factor did not help significantly in the emulsification process.

The zeta potential is a stability indicative parameter in colloidal systems like submicron emulsions [22–24] due to electrostatic repulsion. Figure 2 demonstrated that by decreasing the addition rate and the composition of oil, the zeta potential increased. This observation could be due to the stabilization mechanisms; electrostatic and steric mechanism. At low addition rate and composition of oil, the effect of steric stabilization is caused by the incorporation of surfactant and co-surfactant. However, by increasing the addition rate and oil composition, the surfactant and co-surfactant were unable to increase the electrostatic repulsion between emulsion droplets, as the amount was insufficient. In addition, decreasing the addition rate at high range (10.5–20 mL/min) increased the zeta potential with increasing oil composition. The reason for this behavior could be attributed to the strong repulsive Coulomb force between charged particles which counterbalances the Van der Waals attraction force and this phenomenon is not only contributed by the surfactant role but is also due to the decreasing addition rate.

Polydispersity index (PI) characterizes the disperse systems with respect to deviation from the average size, and values up to 0.250 are acceptable for parenteral emulsions [25]. The polydispersity index varied from 0 to 1. The composition of materials and the preparation method used play an important role in the emulsion formulation as both may affect each other and thus influence the physicochemical properties of the emulsions. Figure 3 demonstrated that the polydispersity index increased with increasing addition rate and Cremophor EL composition at low range (0.5%–0.8% *w*/*w*). The increasing polydispersity index could be due to the high addition rate of the oil phase over the mixture of the aqueous phase, thus limiting the breaking down of the oil droplet during the emulsification process. This promoted the formation of a large particle size. The presence of a high concentration of emulsifier ultimately led to an increase in the flow resistance in the batch emulsification process which in turn resulted in the larger magnitude of apparent viscosity of the prepared emulsions [19,26]. The higher concentration of co-emulsifier produced an emulsion with high viscosity, which can considerably affect the emulsification efficiency. Consequently, this condition increased the coalescence rate resulting in a larger particle size. The large particles tend to coalesce faster than small particles. This phenomenon contributed to the high polydispersity index as the distribution of the particle size became broad. In addition, the low amount of Cremophor EL used, was insufficient to emulsify the emulsion system.

By increasing the addition rate (11–20mL/min) and composition of Cremophor EL (0.85%–1.5% *w*/*w*) the polydispersity index decreased. The decrease in polydispersity index could be due to the co-surfactant, Cremophor EL which possesses polyethylene glycols and ethoxylated glycerol polar covalent parts. Both structures have excellent capability to solubilize many types of essential oil. Because of these two highly hydrophilic groups in the aqueous phase, the difference in viscosity between the two immiscible phases is reduced, thereby leading to lowering of the critical Weber number followed by an increased droplet break-up efficiency [21]. Cremophor EL, which is a non-ionic surfactant, was chosen as co-surfactant not only due to its great emulsifying properties but also due to its characteristically low toxicity.

### 2.4. Optimization of Responses for Formulating Levodopa Nanoemulsions

By using Design-Expert software, the desirability function was probed to acquire an optimized formulation. An optimum levodopa nanoemulsion is that with smallest particle size, lowest polydispersity index and highest zeta potential. The response surface and contour plot were used to visualize the interaction between the independent variables. By investigating the interaction effect between the independent variables and evaluating the optimization constraints, the optimum levodopa nanoemulsion was prepared with a composition of 7.14% oil, 2.2% lecithin, 1.24% Cremophor EL, and an addition rate of 5.5 mL/min. Based on the optimum formulation, the predicted values of particle size, zeta potential and polydispersity index are 104.04 nm, −29.18 mV and 0.136, respectively.

### 2.5. Verification of the Reduced Models

Experimental and predicted values of the responses were compared to check the adequacy of the response surface equations. The optimized formulation of levodopa-loaded nanoemulsion has a particle size of 109.63 nm, zeta potential of −31.06 and polydispersity index of 0.174. As displayed in Table 4, no significant (*p* > 0.05) difference was noted between the experimental and theoretical predicted value. The sufficiency of the corresponding response surface models was verified based on the observations.

### 2.6. Stability Study

**Figure 4:**
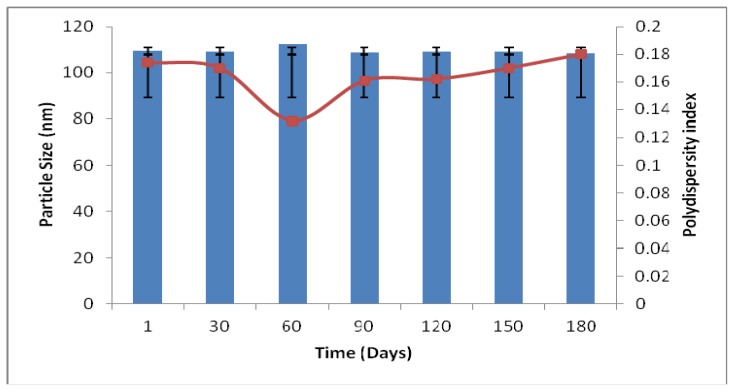
Stability of the optimized levodopa nanoemulsion upon storage at 4 °C as a function of particle size and polydispersity index over storage of 6 months.

For parenteral emulsions, the droplet size and polydispersity index (PI) are important physicochemical parameters since large particle sizes are clinically unacceptable due to emboli formation [22,27]. Figure 4 depicts the particle size and polydispersity index of optimized formulation over time. All levodopa-containing nanoemulsions prepared based on the recommended optimum condition were stable at 4 °C ± 1 °C during the tested period (6 months). No significant changes of particle size and polydispersity index were observed over a period of six months. The excellent stability could be due to the steric stabilizing effect of the non-ionic emulsifier (lecithin) in which a bulk steric barrier is formed against particle collision. Thus, this phenomenon prevents the occurrence of flocculation and coalescence. Improved emulsion stabilization could be explained by the presence of some free emulsifier in micellar form as it plays a vital role in preventing the coalescence after emulsification and storage [20]. Furthermore, it has been reported that MCT can destabilize the emulsion with respect to droplet coalescence, while LCT can increase the viscosity of MCT and the particle size distribution of emulsions, which would increase the stability of emulsions during storage [23]. In essence, it is hypothesized that the whole nano-system is in a stable state and this might be due to the rapid absorption of the non-ionic surfactant, lecithin onto the droplet interface

## 3. Experimental Section

### 3.1. Material

Palm oil was purchased from Sime Darby Jomalina Sdn Bhd, Malaysia. The composition of palm oil is 44.3% palmitic acid, 38.7% oleic acid, 10.5% linoleic acid, 4.6% stearic acid, 1.0% myristic acid, and 0.9% other material which can be considered as impurities. Medium-chain Triglyceride oil was purchased from Pharm-D Sendirian Berhad, Malaysia. Pure soy bean lecithin with 70% phosphatidylcholine (Lipoid S 75) was purchased from Lipoid GmbH, Ludwigshafen-Germany. Polyethylene glycol 400 (PEG 400) was purchased from Merck, USA. Cremophor EL, a non-ionic surfactant with pH of 6.0 to 8.0 was purchased from Sigma-Aldrich, France. Glycerol was purchased from JT Baker, USA. Levodopa was purchased from Noveltek Lifescienceco, Limited, Hong Kong, China. Water was deionised by Milli-Q filtration system.

### 3.2. Formation of Nanoemulsions Containing Levodopa

Nanoemulsions were formulated using a mixture of palm and MCT oil containing levodopa as dispersed oil phase and Mili-Q water as the continuous aqueous phase. Lecithin was dissolved in the oil phase containing a mixture of palm oil with MCT oil (1:1) at 55 °C for 30 min. Levodopa was added to the oil phase and stirred. PEG 400, Cremophor EL and glycerol were dissolved in the deionised water. The preparation was continued by adding the oil phase dropwise to the aqueous solution with continuous stirring using the overhead stirrer (IKA^®^ RW 20 Digital, Nara, Japan) at 300 rpm. The mixture was pre-emulsified with a high shear homogenizer (Kinematica, Lucerne, Switzerland) at 4000 rpm. The pre-emulsification step was performed for 5 min and repeated three times. The resultant coarse emulsion was subjected to a high pressure homogenizer (Gea Niro Soavi S.p.a) for 10 cycles at 800 bars. Table 5 depicts the composition of formulated nanoemulsion.

### 3.3. Experimental Design

A four-factor CCD was utilized to study the effect of oil composition (6%–12% *w*/*w*, A), lecithin composition (1%–3% *w*/*w*, B), Cremophor EL composition (0.5%–1.5% *w*/*w*, C) and addition rate (2–20 mL/min, D) on the three response variables; particle size (R_1_), zeta potential (R_2_) and polydispersity index (R_3_). Hence, based on the CCD, a total of 30 experiments were run using Design Expert software (version 6.0.6, Stat ease Inc, Minneapolis, USA). The experimental runs involved 16 factorial points, 8 axial points and 6 replicates of centre points at 3 levels. The choice of CCD as the experimental design is for the following reasons: it is more precise for estimating factor effects, the interaction effect between factors can be evaluated and permits optimization in the full factor space. The experimental data was analyzed by response surface regression procedure and the results were statistically analyzed by corresponding analysis of variances. An appropriate polynomial model was chosen based on the significant terms (*p* < 0.005), the least significant lack of fit, coefficient of variance, the multiple correlation coefficient, and adjusted multiple correlation coefficient provided by Design-Expert software. The experiments were carried out in randomized order in order to minimize the effect of unexplained variability on the actual response due to extraneous factors [28]. To determine the repeatability of the method, the center point was repeated six times. The quadratic model was obtained in the design of the experimental space and the upper and lower levels are exhibited in Table 6. The CCD matrix is shown in Table 7.

### 3.4. Statistical Analysis

The optimum condition of the independent variables was ascertained by conducting Response Surface Methodology to predict the variation of material compositions as well as preparation conditions. The optimal composition and conditions of preparation of levodopa-loaded nanoemulsions were chosen based on the condition of attaining minimum particle size (*R*_1_), maximum zeta potential (*R*_2_) and minimum polydispersity index (*R*_3_). By using the polynomial regression equation, the response surface behavior was explored for the response function (*Y_i_*). The generalized response surface model is as shown below:

$$\begin{array}{l} Y_{i}=\beta_{0}+\beta_{1}x_{1}+\beta_{2}x_{2}+\beta_{3}x_{3}+\beta_{4}x_{4}-\beta_{11}{x_{1}}^{2}+\beta_{22}{x_{2}}^{2}+\beta_{33}{x_{3}}^{2}+\beta_{44}{x_{4}}^{2}+\beta_{12}x_{1}x_{2}+\beta_{13}x_{1}x_{3}+\beta_{14}x_{1}x_{4}\\ +\beta_{23}x_{2}x_{3}+\beta_{24}x_{2}x_{4}+\beta_{34}x_{3}x_{4} \end{array}$$

Where *Y_i_* is the predicted response; *β*_0_ is constant; *β_i_*, *β_ii_* are the linear, quadratic and interaction coefficients, respectively [21]. The significant differences between the independent variables were determined by utilizing Analysis of Variance (ANOVA). Only the significant (*p* < 0.05) independent variables effects were involved in the reduced model. The non-significant (*p* > 0.005) independent variables were eliminated. Then, the experimental data was refitted to the significant regression coefficient (*p* > 0.05). Consequently, the final reduced model was obtained. Two dimensional contour plots and three dimensional response surface plots were constructed to see the interaction effect of the variables on the responses. It was suggested that for a good fit of a model, *R*^2^ should be at least 0.80 [21].

### 3.5. Verification of Models

Quantitative comparison between the theoretical prediction and obtained experimental values was made to validate the models. In addition, the percentage of the calculated value was also determined. The predicted error is the difference between the experimental value and the predicted value per predicted value [29].

### 3.6. Particle Size and Polydispersity Index

Dynamic light scattering was used to analyze the particle size and polydispersity index of the nanoemulsion by using Malvern Nano ZS90, Malvern, UK. The measurement was performed at a scattering angle of 173° at 25 °C. The nanoemulsions were diluted with deionised water to the required concentration. Then the diluted emulsions were placed in the cuvette. The count rate was maintained between 100 and 300 kcps.

### 3.7. Zeta Potential

Dynamic light scattering was used to measure the zeta potential of the nanoemulsion by using Malvern Nano ZS90, Malvern, UK. The measurement was carried out at a scattering angle of 173° at 25 °C. The nanoemulsions were diluted with deionised water to the required concentration. A folded capillary electrophoresis cell was used to measure the zeta potential. The count rate was maintained between 100 and 300 kcps.

### 3.8. Stability Study

After preparation of the Levodopa-containing nanoemulsion based on the optimized formulation, the nanoemulsion was observed over a period of 6 months at 4 °C or until instability was observed at room temperature, 25 °C. The particle size, polydispersity index and zeta potential were evaluated.

## 4. Conclusions

The current study showed that Response Surface Methodology is a beneficial tool for carrying out the optimization study of levodopa nanoemulsion formulations. The variation of the average particle size, zeta potential and polydispersity index were predicted by employing second order polynomial regression. Generally, the linear effect of Cremophor EL had a significant effect (*p* < 0.05) on the zeta potential and polydispersity index while the addition rate had a significant effect on the particle size and polydispersity index. Conversely, all the responses were significantly (*p* < 0.05) affected by the interaction effect between oil composition and lecithin composition, oil composition and addition rate and between Cremophor EL and addition rate. The quadratics of all four independent variables had significant (*p* < 0.05) effect on the the three response variables studied. The high stability of the Levodopa-loaded nanoemulsion was due to the stabilizing effect of lecithin and Cremophor EL.

## Figures and Tables

**Table 1:** The experimental data obtained for the three responses.

Experiment Number	*R*_1_	*R*_2_	*R*_3_
1	89.49	23.10	0.14
2	101.87	25.40	0.12
3	100.90	24.10	0.12
4	145.40	28.93	0.23
5	101.85	30.42	0.14
6	101.00	23.10	0.13
7	93.54	36.80	0.16
8	104.70	1.19	0.18
9	107.23	2.76	0.15
10	133.25	26.70	0.13
11	103.47	7.67	0.20
12	96.88	33.80	0.19
13	119.70	35.45	0.22
14	106.70	28.70	0.16
15	186.30	25.45	0.13
16	91.39	36.45	0.15
17	100.48	26.35	0.14
18	107.93	29.65	0.15
19	145.50	18.30	0.16
20	99.87	37.00	0.10
21	108.95	23.05	0.16
22	117.50	30.90	0.32
23	113.80	32.00	0.16
24	84.68	36.10	0.17
25	122.80	26.33	0.19
26	105.10	23.37	0.14
27	88.68	35.88	0.16
28	99.50	20.23	0.13
29	99.47	29.43	0.11
30	87.89	26.10	0.19

*R*_1_: Particle Size; *R*_2_: ζ potential; *R*_3_: Polydispersity index.

**Table 2:** Regression coefficients, adjusted and probability values for the final reduced models.

Regression Coefficient	*R*_1_	*R*_2_	*R*_3_
A^0^	102.958	24.802	0.140
A	−3.83	1.938	−0.004
B	−10.623	1.2	−0.0013
C	0.896	5.727	−0.009
D	7.206	−1.245	0.039
A^2^	−0.155	2.36	0.0017
B^2^	5.896	−0.609	−0.0008
C^2^	0.783	−2.622	0.00051
D^2^	0.855	2.564	0.024
AB	−10.3	0.643	−0.0069
AC	−2.081	−1.436	−0.0011
AD	−6.749	3.825	−0.008
BC	2.559	1.628	0.0039
BD	2.674	−2.052	−0.0067
CD	−1.429	−0.885	−0.0157
*R*^2^	0.976	0.909	0.963
*R*^2^ (Adjusted)	0.922	0.671	0.882
Regression (*p*-value)	<0.0001	0.0284	0.0003
Lack of Fit (*p*-value)	0.0514	0.0711	0.2241

A_0_ is constant, A, B, C and D are the linear, quadratic and interaction coefficients of the quadratic polynomial coefficient. *R*_1_: Particle Size; *R*_2_: ζ potential; *R*_3_: Polydispersity index.

**Table 3:** Analysis of variance (ANOVA) of regression coefficient of the fitted quadratic equation.

	Variables	*R*_1_		*R*_2_		*R*_3_	
		
		*F* Value	*p* Value	*F* Value	*p* Value	*F* Value	*p* Value
Main effects	A	3.393	0.0986	1.087	0.3277	0.606	0.4564
	B	78.41	<0.0001	0.417	0.5366	0.201	0.6648
	C	0.558	0.474	9.492	0.0151	9.242	0.014
	D	12.028	0.0071	0.449	0.5218	58.543	<0.0001

Quadratic effects	A^2^	0.019	0.8929	5.527	0.0466	0.404	0.541
	B^2^	27.606	0.0005	0.368	0.561	0.102	0.7567
	C^2^	0.487	0.5027	6.823	0.031	0.034	0.8573
	D^2^	0.581	0.4655	6.522	0.034	75.862	<0.0001

Interaction effects	AB	49.143	<0.0001	0.24	0.6377	3.611	0.0898
	AC	2.006	0.1904	1.193	0.3065	0.091	0.7703
	AD	21.101	0.0013	8.467	0.0196	5.114	0.0501
	BC	3.034	0.1155	1.535	0.2505	1.155	0.3104
	BD	3.034	0.1021	2.438	0.1571	3.418	0.0975
	CD	3.034	0.3562	0.454	0.5195	18.856	0.0019

A: Composition of oil; B: Composition of Lecithin, C: Composition of Cremophor EL; D: Addition rate. *R*_1_: Particle Size; *R*_2_: Zeta potential; *R*_3_: Polydispersity index.

**Table 4:** The predicted and observed response values for the optimized nanoemulsion.

Response	Predicted	Observed
*R*_1_: Particle size	104.04	109.63
*R*_2_: Zeta potential	−29.18	−31.06
*R*_3_: Polydispersity index	0.136	0.174

**Table 5:** Composition of oil and aqueous phase formulated nanoemulsion.

Materials	Amount (*w*/*w*, %)
*Oil phase*	
Palm Oil	5
MCT Oil	5
Lecithin	3
Levodopa	0.9

*Aqueous phase*	
Polyethylene glycol 400	0.45
Cremophor EL	0.4
Glycerol	2
Deionised water q.s	100

**Table 6:** Levels of independent variables in central composite design (CCD).

Independent variables	Coded Levels				

	Axial (−α)	Low	Centre	High	Axial (+α)
Palm oil: MCT oil (1:1) (%, *w*/*w*)	3	6	9	12	15
Lecithin (%, *w*/*w*)	0	1	2	3	4
Cremophor EL (%, *w*/*w*)	0	0.5	1	1.5	2
Addition rate (mL/min)	−7	2	11	20	29

**Table 7:** The matrix of central composite design (CCD).

Experiment Number	Blocks	A	B	C	D
1 *	Block 1	9	2	1	11
2	Block 1	12	3	0.5	20
3	Block 1	12	3	1.5	2
4	Block 1	6	3	0.5	2
5	Block 1	6	1	0.5	20
6	Block 1	12	1	1.5	20
7 *	Block 1	9	2	1	11
8	Block 1	6	3	1.5	20
9	Block 1	12	1	0.5	2
10	Block 1	6	1	1.5	2
11	Block 2	6	3	1.5	2
12 *	Block 2	9	2	1	11
13	Block 2	12	3	1.5	20
14	Block 2	6	1	1.5	20
15	Block 2	12	1	1.5	2
16	Block 2	12	3	0.5	2
17 *	Block 2	9	2	1	11
18	Block 2	6	3	0.5	20
19	Block 2	12	1	0.5	20
20	Block 2	6	1	0.5	2
21 *	Block 3	9	2	1	11
22	Block 3	9	2	0	11
23	Block 3	15	2	1	11
24	Block 3	9	2	1	−7
25 *	Block 3	9	2	1	11
26	Block 3	9	4	1	11
27	Block 3	9	2	2	11
28	Block 3	9	0	1	11
29	Block 3	3	2	1	11
30	Block 3	9	2	1	29

A: Composition of oil; B: Composition of Lecithin, C: Composition of Cremophor EL; D: Addition rate.

* Center point.
